# Illuminating the landscape of high-level clinical trial opportunities in the *All of Us* Research Program

**DOI:** 10.1093/jamia/ocae062

**Published:** 2024-04-15

**Authors:** Cathy Shyr, Lina Sulieman, Paul A Harris

**Affiliations:** Department of Biomedical Informatics, Vanderbilt University Medical Center, Nashville, TN 37203, United States; Department of Biomedical Informatics, Vanderbilt University Medical Center, Nashville, TN 37203, United States; Department of Biomedical Informatics, Vanderbilt University Medical Center, Nashville, TN 37203, United States; Department of Biostatistics, Vanderbilt University Medical Center, Nashville, TN 37203, United States; Department of Biomedical Engineering, Vanderbilt University, Nashville, TN 37240, United States

**Keywords:** clinical trial diversity, electronic health records, social determinants of health, phenome-wide association study, *All of Us* Research Program

## Abstract

**Objective:**

With its size and diversity, the *All of Us* Research Program has the potential to power and improve representation in clinical trials through ancillary studies like *Nutrition for Precision Health*. We sought to characterize high-level trial opportunities for the diverse participants and sponsors of future trial investment.

**Materials and Methods:**

We matched *All of Us* participants with available trials on ClinicalTrials.gov based on medical conditions, age, sex, and geographic location. Based on the number of matched trials, we (1) developed the Trial Opportunities Compass (TOC) to help sponsors assess trial investment portfolios, (2) characterized the landscape of trial opportunities in a phenome-wide association study (PheWAS), and (3) assessed the relationship between trial opportunities and social determinants of health (SDoH) to identify potential barriers to trial participation.

**Results:**

Our study included 181 529 *All of Us* participants and 18 634 trials. The TOC identified opportunities for portfolio investment and gaps in currently available trials across federal, industrial, and academic sponsors. PheWAS results revealed an emphasis on mental disorder-related trials, with anxiety disorder having the highest adjusted increase in the number of matched trials (59% [95% CI, 57-62]; *P* < 1e-300). Participants from certain communities underrepresented in biomedical research, including self-reported racial and ethnic minorities, had more matched trials after adjusting for other factors. Living in a nonmetropolitan area was associated with up to 13.1 times fewer matched trials.

**Discussion and Conclusion:**

*All of Us* data are a valuable resource for identifying trial opportunities to inform trial portfolio planning. Characterizing these opportunities with consideration for SDoH can provide guidance on prioritizing the most pressing barriers to trial participation.

## Introduction

Improving clinical trial participation among diverse populations is crucial for advancing equitable health research. Over the last 30 years, promoting diversity and inclusion in clinical trials emerged as a key priority for U.S. federal agencies, such as the National Institutes of Health (NIH) and the Food and Drug Administration.^1^^,^^2^ Despite efforts to address long-standing inequities in trial accrual, little progress has been made to increase participation among historically marginalized populations (eg, minoritized racial and ethnic groups, older adults, rural residents, and women).^3^

A promising strategy is recruitment from ongoing large-scale health cohorts. Successful nationwide trials like VITAL^4^ and COSMOS^5^ that enrolled participants from the Women’s Health Study^6^ and Women’s Health Initiative,^7^ respectively, demonstrated the potential of embedded recruitment to enable timely accrual of participants who have largely and historically been underrepresented in biomedical research. Specifically, underrepresented groups in current health cohort studies have already demonstrated interest and willingness to participate in health-related research, reducing key barriers such as mistrust and lack of awareness.^8^ In addition, the availability of participant-level data, such as routine demographics and electronic health records (EHRs), can help investigators quickly identify potential participants from diverse populations to accelerate start-up times and shorten enrollment phases.^9^

The *All of Us* Research Program is an ongoing national initiative with the potential to inform and power clinical trials through ancillary studies aimed at returning value to the Program participants and enriching the *All of Us* data.^10^ Created following the announcement of the U.S. Precision Medicine Initiative in 2015, the Program seeks to enroll at least 1 million participants to help build one of the most diverse biomedical research repositories in the world. Since its inception, the *All of Us* Research Program has prioritized recruitment of participants from communities historically underrepresented in biomedical research, including minority racial and ethnic groups, sexual and gender minorities, older adults, people with disabilities, people with barriers in access to care, people with low income or educational attainment, and rural residents.^11^ The Program’s community and engagement partners focus on educating communities and supporting enduring relationships with participants.^12^ As a result, more than 80% of participants come from communities historically underrepresented in biomedical research, making the *All of Us* dataset the most diverse of its kind.^13^^,^^14^ In 2022, the NIH awarded $170 million for *Nutrition for Precision Health, powered by the All of Us Research Program*, an ancillary study that began enrolling *All of Us* participants for precision nutrition studies, including interventional clinical trials, on March 30, 2023.^15^ Ancillary studies like *Nutrition for Precision Health* will return value by generating new biomedical data accessible to the wider research community, providing engagement opportunities for underrepresented groups to participate in clinical trials, and enabling long-term discoveries to improve health for diverse populations. Thus, sponsors of future ancillary studies and participants of the *All of Us* Research Program represent assets that are capable of advancing equitable health research. The Program’s potential to power a wide range of clinical trials, however, has not been studied to date. Furthermore, opportunities for sponsors of future trial investment are currently under-explored.

To this end, the objective of this study is to characterize at a high level the availability of recruiting clinical trials in the U.S. for the *All of Us* participant population. Our work aims to return value to communities through several ways. First, illuminating high-level trial availability can help sponsors evaluate portfolios and identify potential investment opportunities in the current clinical trial landscape to inform policy making and planning for *All of Us* ancillary studies. These studies will not only catalyze short-term engagement opportunities for *All of Us* participants, but also enable long-term health discoveries. Second, characterizing high-level trial opportunities with consideration for social determinants of health (SDoH) can identify barriers to trial participation. Assessing the interplay between trial opportunities and socioenvironmental factors in a national health cohort like *All of Us* can shed light on these barriers across diverse populations. Third, our flexible, data-driven approach can readily generalize to other large-scale cohorts to illuminate the trial landscape for the broader scientific community.

## Methods

### Study design and participants

**Figure 1.**
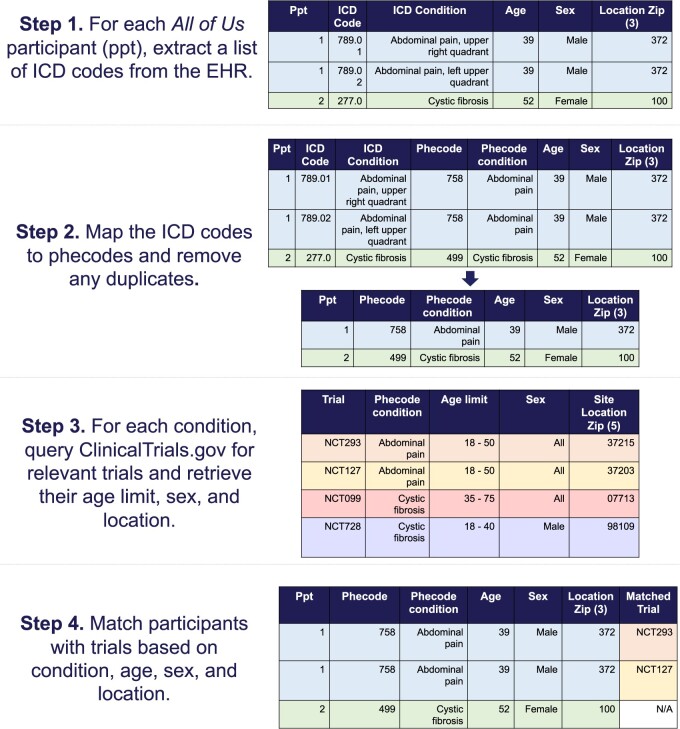
Overview of matching procedure.

This cross-sectional study included *All of Us* participants with a 3-digit zip code and at least one condition in their EHR from the *All of Us* June 2022 Controlled Tier dataset. The presence of a condition was defined as having at least 2 instances of a relevant International Classification of Diseases (ICD) code to maximize phenotyping accuracy.^16^^,^^17^ We grouped ICD codes into phecodes (version 1.2),^18^ which are physician-curated codes intended to capture clinically meaningful concepts or conditions. For each condition, we used ClinicalTrials.gov’s application programming interface (API) (v1.01.05) (https://clinicaltrials.gov/api/gui) to query study records. We restricted our query to actively recruiting (as of February 14, 2023), U.S.-based studies, and retrieved the following fields: the study’s unique identifier, age limit, sex at birth, location, study type, and lead sponsor information. We identified relevant adult trials with the study type “interventional” and minimum age ≥18 years. Figure 1 provides a schema detailing the query and matching process. A description of each field as defined on ClinicalTrials.gov and a sample API query are provided in Table S1. The *All of Us* Controlled Tier dataset has a nonhuman subjects designation, and ClinicalTrials.gov data are publicly available, aggregate trial data; thus, institutional review board approval was not required. This study followed the Strengthening the Reporting of Observational Studies in Epidemiology (STROBE) guideline.^19^

### Characterization of trial opportunities for *All of Us* participants and sponsors of future trial investment

For every *All of Us* participant, we calculated the number of matched trials based on medical conditions, age, sex at birth, and geographic location (3-digit zip code) (Figure 1). To help sponsors navigate the current trial landscape, we developed the Trial Opportunities Compass (TOC), a generalizable, data-driven framework that identifies opportunities for future portfolio investment and gaps in currently available trials. Specifically, the TOC divides medical conditions into 1 of 9 regions, each characterized by a combination of: (1) few, (2) some, and (3) many participants/matched trials (ie, a 3x3 grid). Because sponsors can have different portfolio investment interests, the TOC is designed so that users can specify the cut-offs for these regions (eg, <20 percentile=few, between 20 and 80 percentile=some, >80 percentile=many). The rationale for having a gradient of regions is to help sponsors quickly and easily identify potential opportunities or gaps in the trial landscape. For example, conditions that belong to the region with “many participants and few trials” (ie, prevalent but under-studied conditions) represent potential trial investment opportunities, whereas those that belong to “few participants and many trials” (ie, less prevalent but well-studied conditions) may correspond to saturated research areas. To characterize the trial opportunities landscape in the Program, we conducted a phenome-wide association study (PheWAS) to assess the relationship between the number of matched trials and medical conditions. Specifically, we sought to characterize the diversity of trial investment portfolios in the current landscape and identify conditions that were associated with more matched trials, representing trial opportunities. Identifying trial opportunities can help guide sponsors on resource allocation and portfolio planning. In a secondary analysis, we assessed the relationship between the number of matched trials and SDoH to better understand the interplay between trial opportunities and socioenvironmental factors across the diverse participant population.

### Statistical analysis

We calculated the average number of matched trials stratified by age, sex at birth, self-reported race and ethnicity, geographic location, highest educational level attained, and income level across different phecode condition domains. For geographic location, we categorized participants into living in a metropolitan or nonmetropolitan area according to the U.S. Department of Agriculture^20^ based on their 3-digit zip code. For income status, participants were categorized into the low-income group if their annual household income did not exceed the low-income limit defined by the U.S. Department of Housing and Urban Development based on household size, 3-digit zip code, and fiscal year.^21^ For our PheWAS, we performed multivariable negative binomial regressions to assess the association between the number of matched trials (outcome) and the presence of each medical condition (present or absent) adjusting for age, sex at birth, location, self-reported race and ethnicity, highest educational level attained, income status (low income vs not low income), disability (have one or more disability vs no disability), and number of medical conditions. We chose the negative binomial distribution to account for overdispersion in the outcome data and used Bonferroni’s correction to adjust for multiple testing based on a significance threshold of 0.05. For our demographic and SDoH analysis, we used multivariable negative binomial regression to assess the association between the number of matched trials and various factors, including demographics (self-reported race and ethnicity, sex at birth, age), socioenvironmental factors (geographic location, income status, highest educational level attained, English proficiency, food insecurity, discrimination, social support, housing quality, and neighborhood cohesion),^22^ and number of medical conditions. All statistical analyses were performed using R version 4.2.2 on the *All of Us* Researcher Workbench.^23^

## Results

The June 2022 release of the *All of Us* Controlled Tier data included 210 491 participants with at least one medical condition in their EHR; among those, 181 529 had a 3-digit zip code. Based on our ClinicalTrials.gov query from February 14, 2023, there were 18 634 actively recruiting, adult trials with at least one U.S.-based recruiting site. The majority (95%) of these trials had at least one recruiting site in a metropolitan area, whereas only 19% had a recruiting site in a nonmetropolitan area. The phecode condition domains with most number of trials were mental, cancer, neurological, endocrine/metabolic, circulatory, and respiratory. To ensure legibility of the table/figures, we consolidated the rest of the domains as “other.”

### Overview of trial opportunities in the *All of Us* research program

**Figure 2.**
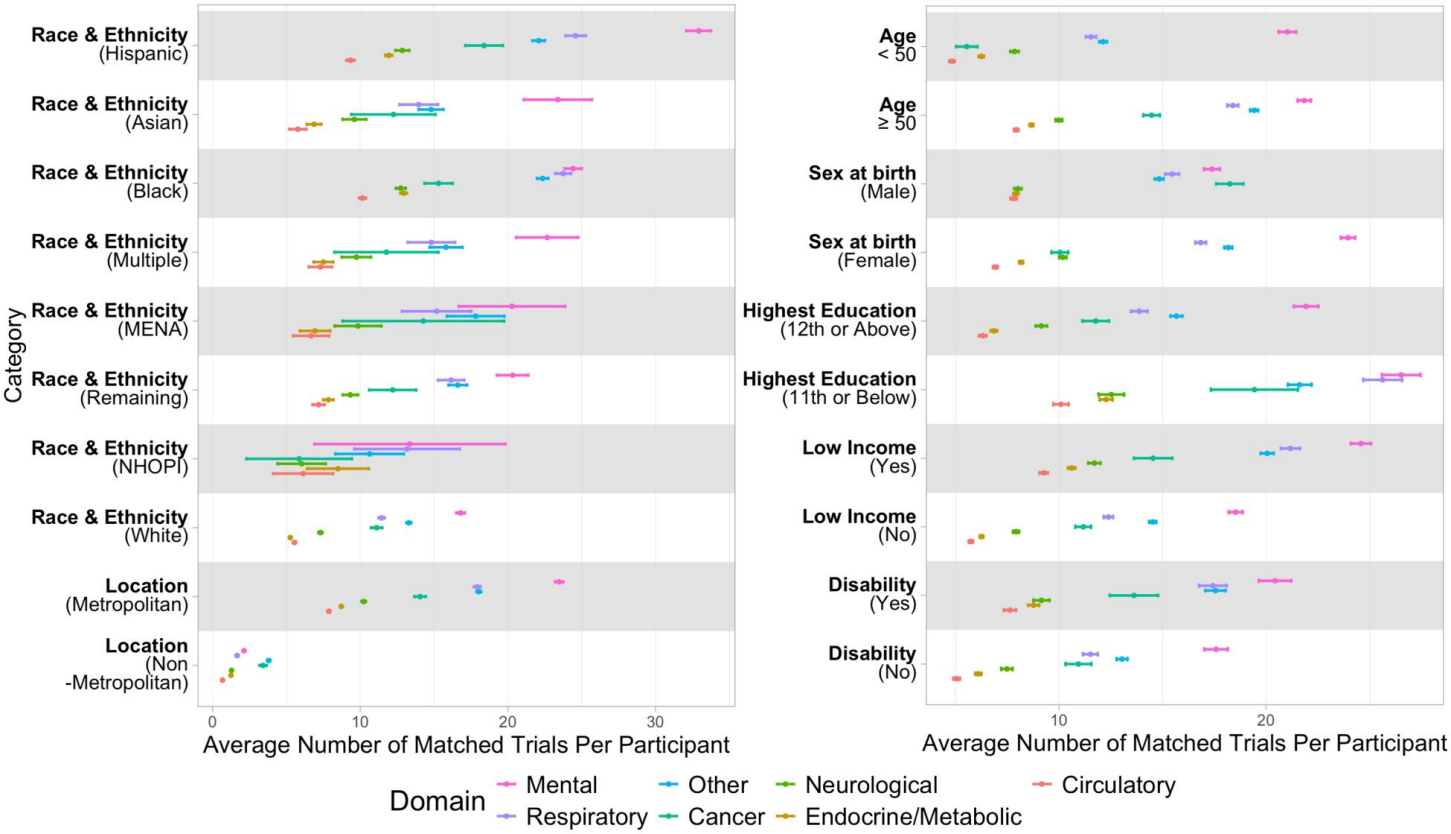
Average number of matched trials and 95% CI per *All of Us* participant by sociodemographic factors. Within each category, the dots and horizontal bars represent the average number of matched trials and 95% CI, respectively. These are color coded by phecode-derived medical domains: Mental (pink), Respiratory (purple), Other (blue), Cancer (green), Neurological (dark green), Endocrine/Metabolic (gold), and Circulatory (red). The phecode domain “Other” consists of digestive, hematopoietic, sense organs, symptoms, musculoskeletal, genitourinary, injuries & poisoning, pregnancy complications, dermatologic, congenital anomalies, and infectious diseases. Self-reported race and ethnicity categories: Hispanic = Hispanic, Latino or Spanish; Black = Black, African American, or African; MENA = Middle Eastern or North African; NHOPI = Native Hawaiian or other Pacific Islander. The self-reported race and ethnicity category “Remaining” consists of participants who skipped the survey question or selected “prefer not to answer.”

Table 1 provides an overview of the cohort across sociodemographic factors and condition domains. The majority of the cohort was ages 50 years or above (65%), female (64%), self-reported white (52%), and lived in metropolitan areas (93%). Mental, neurological, circulatory, endocrine/metabolic, and respiratory conditions were common, with at least 50% of participants having at least one corresponding phecode entry in their EHR. Overall, the average number of matched trials was 58.4 [95% CI, 57.9-58.9] per participant and higher among self-reported racial and ethnic minorities (72.3 [95% CI, 71.5-73.1]). Figure 2 shows the average number of matched trials stratified by sociodemographic factors across 7 condition domains. Participants from communities historically underrepresented in biomedical research, including self-reported Black and Hispanic participants, participants with low income, participants with one or more disabilities, and participants without a high school diploma or equivalent, had more matched trials compared to their respective counterparts across all condition domains. Conversely, participants living in nonmetropolitan areas had substantially fewer matched trials (ie, 3.9-12.2 times fewer) than those in metropolitan areas. Within each sociodemographic factor, participants generally matched with more mental health-related trials than those from other condition domains.

**Table 1.** Overview of demographic, social, and condition profiles.

	*N* (%)^a^
Self-reported demographic and social factors	
Age	
<50	63 727 (35)
≥50	117 802 (65)
Sex at birth	
Male	63 951 (36.2)
Female	112 730 (63.8)
Intersex	37 (<1)
Race and ethnicity	
White	94 814 (52.2)
Black, African American, or African	35 953 (19.8)
Hispanic, Latino, or Spanish	34 693 (19.1)
Asian	4382 (2.41)
Middle Eastern or North African	950 (0.52)
Native Hawaiian or other Pacific Islander	207 (0.11)
Multiple	2671 (1.5)
Remaining	7859 (4.3)
Geographic location	
Metropolitan	167 920 (92.5)
Nonmetropolitan	13 609 (7.5)
Highest educational level attained	
11 or below	17 611 (10.1)
12 or GED	84 354 (48.2)
College graduate	38 424 (21.9)
Advanced degree	34 609 (19.8)
Have a disability	
Yes	14 754 (30.2)
No	34 113 (69.8)
Low income	
Yes	49 584 (35.2)
No	91 318 (64.8)
Condition domains^b^	
Cancer	60 919 (33.8)
Mental	89 826 (50)
Neurological	94 810 (52.6)
Circulatory	111 720 (62)
Endocrine/metabolic	117 157 (65)
Respiratory	99 887 (55.4)
Other	166 538 (92.4)

a *N* (%) denotes the number of participants (percentage).

b Phecode condition domains where participants with at least one corresponding phecode were counted. Note that a participant can be counted towards multiple condition domains. The condition domain “Other” consists of digestive, hematopoietic, sense organs, symptoms, musculoskeletal, genitourinary, injuries & poisoning, pregnancy complications, dermatologic, congenital anomalies, and infectious diseases. The self-reported race and ethnicity category “Remaining” consists of participants who skipped the survey question or selected “prefer not to answer.”

### TOC for navigating the current trial landscape

**Figure 3.**
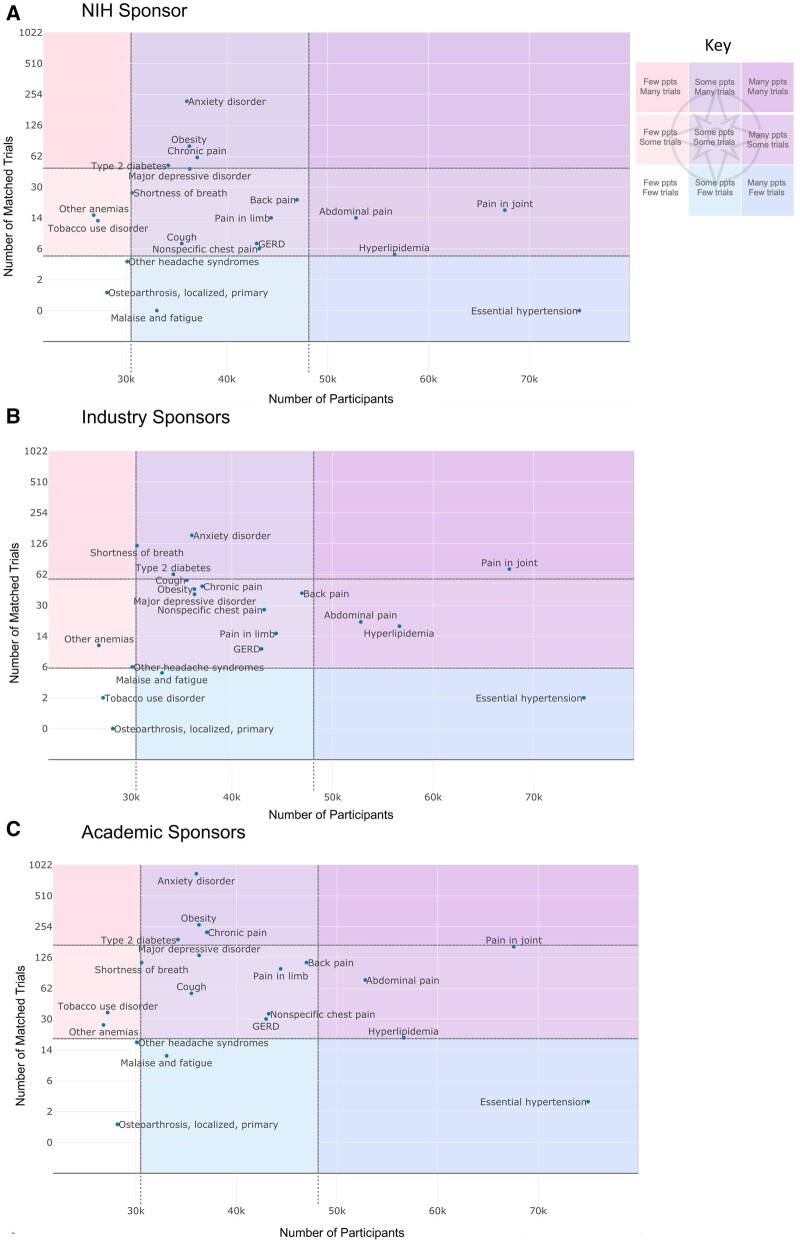
Trial Opportunities Compass for different sponsors across the most common medical conditions in *All of Us*. *Ppts = participants; {few, some many} = {lowest 20th, middle 60th, and top 20th percentiles}*.

Figure 3 shows the TOC for NIH, industrial, and academic sponsors across the most common medical conditions in *All of Us*. For illustration, we divided the landscape into {few, some, many} participants/matched trials based on {the lowest 20th, middle 60th, and top 20th} percentile cutoffs. Overall, the distribution of medical conditions in the TOC is comparable across all sponsors, revealing similar focal areas in their trial portfolio investment. Essential hypertension, hyperlipidemia, and abdominal pain are among the most common medical conditions (many participants), representing potential trial investment opportunities, but under-studied (few to some matched trials) by all 3 sponsors compared to other medical conditions, including anxiety disorder and type 2 diabetes (many matched trials). In particular, anxiety disorder had the most number of matched trials, with 217 by the NIH, 152 industry, and 835 academia. Type 2 diabetes had 50, 62, and 189 matched trials across the NIH, industrial, and academic sponsors, respectively.

### PheWAS of the trial opportunities landscape

**Figure 4.**
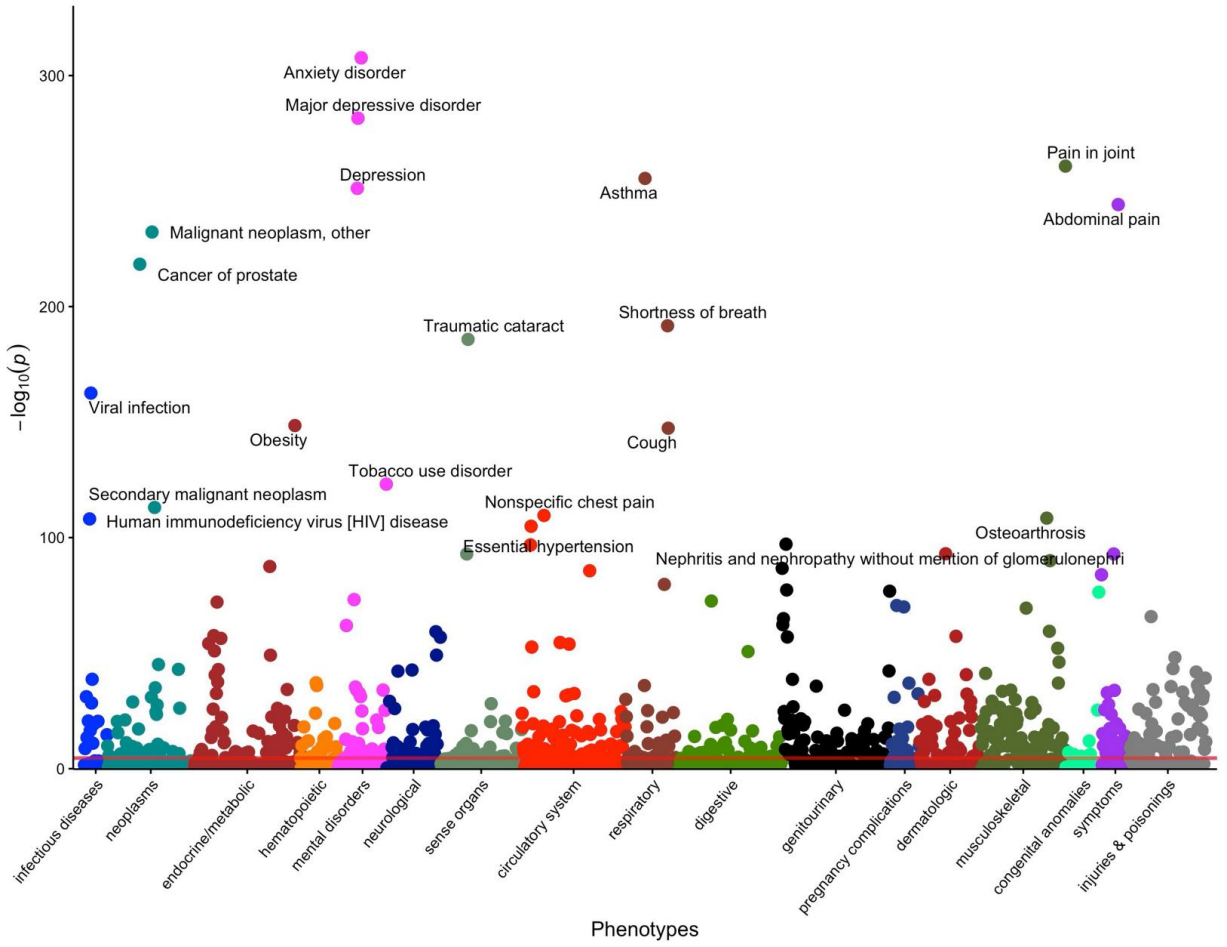
Manhattan plot of the trial opportunities PheWAS for all participants. *N* = 181 529 participants. Red horizontal line indicates the threshold for statistical significance.

Figure 4 shows the Manhattan plot for the entire *All of Us* participant population. For legibility, we labeled the 20 most statistically significant medical conditions. Overall, the PheWAS results revealed a wide range of trial opportunities across different clinical domains. Specifically, we discovered an emphasis on mental disorder-related trials in the current landscape, as 4 of the top 20 hits belonged to this domain. Adjusting for age, sex at birth, location, self-reported race and ethnicity, education, income, disability, and the number of medical conditions, all 4 were significantly associated with more matched trials, with anxiety disorder having the highest increase (ie, 59% [95% CI, 57-62]; *P* < 1e-300) followed by depression (42% [95% CI, 39-44]; *P* = 6e-252), major depressive disorder (34% [95% CI, 32-37]; *P* = 3e-282), and tobacco use disorder (24% [95% CI, 22-26]; *P* = 7e-124). Other focal areas included neoplasms and respiratory conditions. Specifically, malignant neoplasms and secondary malignant neoplasms were associated with a 158% (95% CI, 144-174; *P* = 5e-233) and 122% (95% CI, 107-138; *P* = 8e-114) increase in the number of matched trials, respectively, followed by prostate cancer (100% [95% CI, 91-108]; *P* = 4e-219). Among respiratory conditions, asthma was associated with the highest increase (37% [95% CI, 35-40]; *P* = 3e-256), followed by shortness of breath (30% [95% CI, 29-33]; *P* = 2e-192), and cough (25% [95% CI, 23-27]; *P* = 4e-128).

### Relationship between trial opportunities and socioenvironmental factors

**Figure 5.**
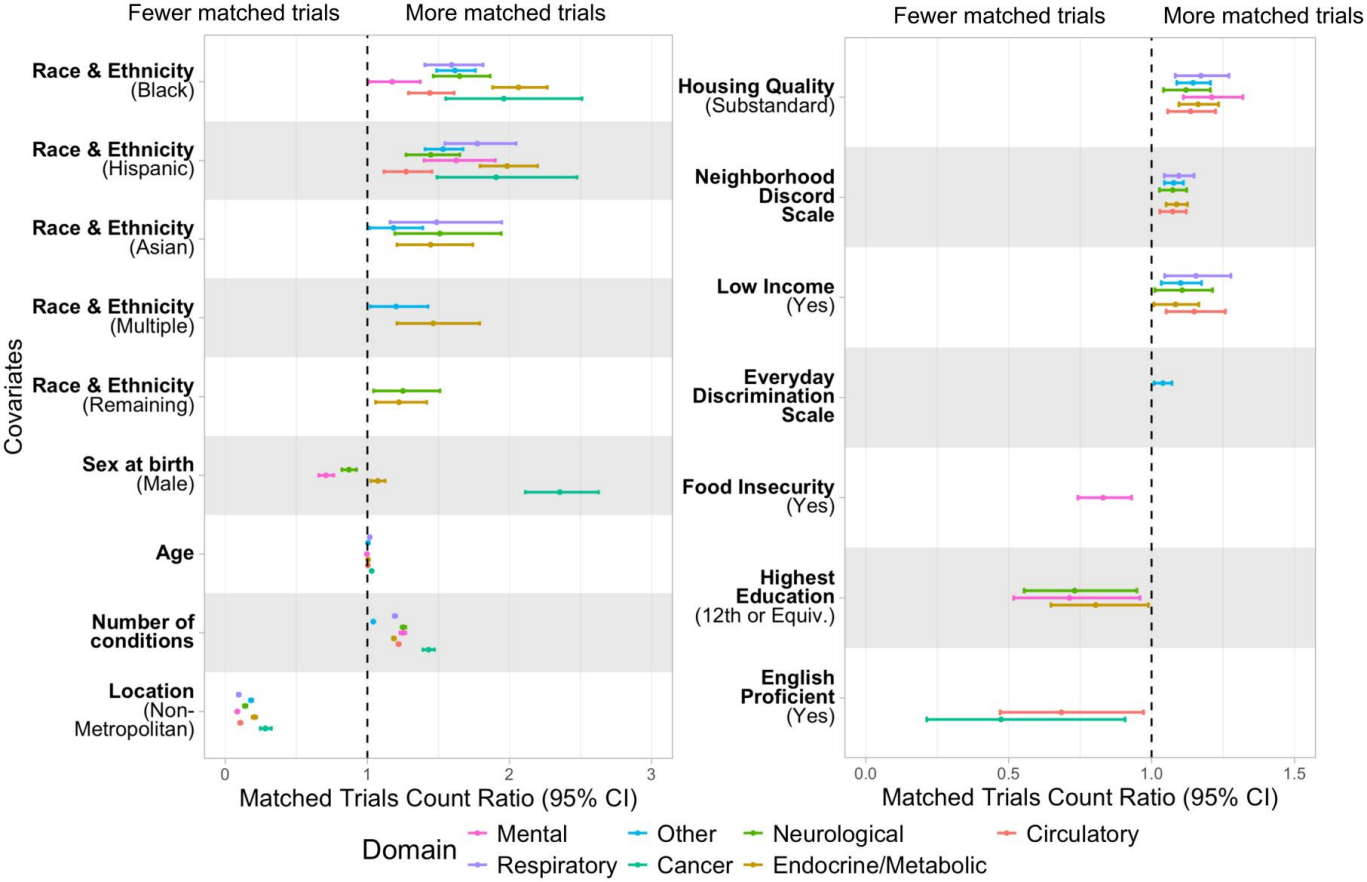
Matched trials count ratio (95% CI) of statistically significant covariates in the adjusted analysis. Reference levels of categorical covariates include: *Self-reported race and ethnicity (White), Sex at birth (Female), Location (Metropolitan), Housing Quality (Good), Low Income (No), Food Insecurity (No), Highest Education (11 or Below), and English Proficient (No).* The domain “Other” consists of digestive, hematopoietic, sense organs, symptoms, musculoskeletal, genitourinary, injuries & poisoning, pregnancy complications, dermatologic, congenital anomalies, and infectious diseases. Self-reported race and ethnicity categories: Hispanic = Hispanic, Latino or Spanish; Black = Black, African American, or African; MENA = Middle Eastern or North African; NHOPI = Native Hawaiian or other Pacific Islander. The self-reported race and ethnicity category “Remaining” consists of participants who skipped the survey question or selected “prefer not to answer.”

Figure 5 shows the statistically significant adjusted matched trial ratios from our demographic and SDoH analysis. Living in a metropolitan area was associated with substantially more matched trials across all condition domains, with metropolitan participants matching with 3.1-13.1 times more trials than their nonmetropolitan counterparts, adjusting for other factors. Compared to self-reported white participants, Black and Hispanic participants had more matched trials across all condition domains, with endocrine/metabolic trials having the highest adjusted ratio (1.65 times more [95% CI, 1.46-1.86] *P* = 3.5e-16 and 1.45 [95% CI, 1.27-1.65] *P* = 2.19e-8, respectively). Substandard housing quality, more neighborhood discord, low income, lower educational attainment, and limited English proficiency were associated with more matched trials across multiple condition domains after adjusting for other factors.

## Discussion

Our high-level analysis illuminated potential opportunities in the current clinical trial landscape. The PheWAS enabled discovery of a wide range of trial opportunities for *All of Us* participants, reflecting the diversity of trial investment portfolios. Specifically, among the 20 conditions with the strongest statistical associations, half were mental disorders, cancers, or respiratory conditions. Our adjusted analysis showed that self-reported racial and ethnic minorities and participants with certain SDoH, ie, environmental stressors and limited healthcare access, had more clinical trial opportunities in the current landscape. It is worth noting, however, that trial availability is not the same as trial accessibility. Trials may not be truly accessible to underrepresented communities due to various reasons, including under-designed access logistics and lack of investment in community engagement.^24^ Thus, significant investment in concurrent initiatives is required to increase trial accessibility among marginalized communities.^3^

While prior work characterized the clinical trial landscape by analyzing trends in trial completion, participant enrollment, and focal areas,^25–27^ ours is the first to pair ClinicalTrials.gov studies with data from a large-scale health cohort to return value to communities by illuminating opportunities for trial planning. Specifically, the TOC can be applied to combinations of different sponsors and medical conditions to assess broad-to-specific scientific portfolios. As such, it may provide meaningful information to inform *All of Us* ancillary study design planning and policy making for sponsors interested in embedded recruitment from large-scale health cohorts. Based on our adjusted analysis on demographic factors and SDoH, living in a nonmetropolitan area was a major limiting factor associated with up to 13.1 times fewer matched trials. This finding supports the observation from qualitative studies that geographic location is a key barrier to trial participation.^28–32^ Therefore, quantifying the magnitude of potential barriers by leveraging a diverse, large-scale health cohort like *All of Us* not only can supplement qualitative studies, but also return value to the biomedical community by providing guidance on prioritizing the most pressing barriers.

Our study has several strengths and limitations. Because phecodes rely on data that are ubiquitous, standardized, and easy to manipulate,^16^ our approach is readily generalizable to other large-scale health cohorts that contain participant-level EHR data. Limitations of phecodes, or ICD-based phenotyping in general, include incomplete or incorrect ascertainment of medical conditions. As a result, our analysis may have missed conditions. While our study focuses on providing a high-level characterization of the national clinical trial landscape based on a broad matching strategy, we recognize that sponsors may also be interested in matching participants based on more granular trial criteria. Specifically, using a broad matching strategy can be helpful for sponsors that need to identify a large group of potential participants; on the other hand, others may prefer a granular matching strategy to identify participants with specific traits (eg, specific genetic markers). Therefore, the definition of a trial opportunity depends on the sponsors’ interests and consequently, their matching strategy. To this end, our framework can be easily tailored to different matching strategies. For sponsors interested in a more granular characterization of the trial landscape, a potential approach is to leverage information from multiple EHR data types, such as lab results and clinical notes, to increase the accuracy and granularity of phenotypes.^33–35^ Natural language processing models that can extract computable phenotypes from ClinicalTrials.gov data may serve as a useful tool for future work in this direction.^36–38^

Our findings support the potential of the *All of Us* Research Program to power a wide range of clinical trials. The diversity and infrastructure of the Program can catalyze much-needed improvement in clinical trial participation from underrepresented populations through ancillary studies like *Nutrition for Precision Health*. As such, these studies provide opportunities for participants and sponsors to address long-standing inequities in trial accessibility and recruitment. This requires building trust between participants and researchers, promoting fairness for participants and their communities, and generating unbiased biomedical knowledge. Ultimately, reaching this goal will take all of us.

## Supplementary Material

ocae062_Supplementary_Data

## Data Availability

To ensure privacy of *All of Us* participants, deidentified data used for this study are available to approved researchers following registration, completion of ethics training, and attestation of a data use agreement through the *All of Us* Research Program website, which can be accessed at https://workbench.researchallofus.org/login. The code used to produce the results in this article can be accessed at https://github.com/cathyshyr/AllOfUs_SDOH_Clinical_Trials.
