# Supplementary material for: Illuminating the landscape of high-level clinical trial opportunities in the All of Us Research Program
Source: J Am Med Inform Assoc. 2024 Apr 15;31(12):2890–8. doi: 10.1093/jamia/ocae062 (PMC11631138; doi:10.1093/jamia/ocae062)
Supplement: ocae062_Supplementary_Data [file ocae062_supplementary_data.docx]

**Supplementary Materials**

**eTable 1. Description of ClinicalTrials.gov fields and sample API query used in this study.**

| **Sample API Query** | **Description** |
| --- | --- |
| https://www.clinicaltrials.gov/api/query/study_fields?expr= hyperlipidemia +SEARCH%5BLocation%5D%28AREA%5BLocationCountry%5DUnited+States+AND+AREA%5BLocationStatus%5DRecruiting%29+&fields=NCTId%2CGender%2CMinimumAge%2CMaximumAge%2CLocationZip%2CLeadSponsorClass%2CStudyType&min_rnk=1&max_rnk=1000&fmt=json" | Queries ClinicalTrials.gov for studies on “hyperlipidemia” in the United States that are actively recruiting and retrieves the fields, NCTId, Gender, MinimumAge, MaximumAge, LocationZip, LeadSponsorClass, and StudyType, for up to 1,000 records* in JSON format  *In our study, we repeated this query to retrieve up to 5,000 records. |
| **Field** |  |
| NCTId | Unique clinical study identifier |
| Sex | The sex and, if applicable, gender of the participants eligible to participate in the clinical study |
| MinimumAge | The numerical value, if any, for the minimum age a potential participant must meet to be eligible for the clinical study |
| MaximumAge | The numerical value, if any, for the maximum age a potential participant must meet to be eligible for the clinical study |
| LocationZip | Zip code of participating facility in the clinical study |
| LeadSponsorClass | The entity or the individual who is the sponsor of the clinical study (NIH, Industry, Individual, Federal, Network, Unknown, Other) |
| StudyType | The nature of the investigation or investigational use for which clinical study information is being submitted (Interventional or Observational) |
